# Emerging healthy lifestyle factors and all-cause mortality among people with metabolic syndrome and metabolic syndrome-like characteristics in NHANES

**DOI:** 10.1186/s12967-023-04062-1

**Published:** 2023-04-01

**Authors:** Mengying Niu, Jiahao Chen, Rongyao Hou, Yu Sun, Qi Xiao, Xudong Pan, Xiaoyan Zhu

**Affiliations:** 1 Department of Neurology, The Affiliated Hospital of Qingdao University, Qingdao, China; 2 Department of Epidemiology and Health Statistics, The School of Public Health of Qingdao University, Qingdao, China; 3 Department of Neurology, The Affiliated Hiser Hospital of Qingdao University, Qingdao, China; 4 Department of Critical Care Medicine, The Affiliated Hospital of Qingdao University, Qingdao, China

**Keywords:** NHANES, Metabolic syndrome, Healthy lifestyle, Mortality

## Abstract

**Background:**

The impact of integrated lifestyles on health has attracted a lot of attention. It remains unclear whether adherence to low-risk healthy lifestyle factors is protective in individuals with metabolic syndrome and metabolic syndrome-like characteristics. We aimed to explore whether and to what extent overall lifestyle scores mitigate the risk of all-cause mortality in individuals with metabolic syndrome and metabolic syndrome-like characteristics.

**Methods:**

In total, 6934 participants from the 2007 to 2014 National Health and Nutrition Examination Survey (NHANES) were included. The weighted healthy lifestyle score was constructed based on smoking, alcohol consumption, physical activity, diet, sleep duration, and sedentary behavior information. Generalized linear regression models and restricted cubic splines were used to analyze the association between healthy lifestyle scores and all-cause mortality. ​

**Results:**

Compared to participants with relatively low healthy lifestyle scores, the risk ratio (RR) in the middle healthy lifestyle score group was 0.51 (RR = 0.51, 95% CI 0.30–0.88)*,* and the high score group was 0.26 (RR = 0.26, 95% CI 0.15–0.48) in the population with metabolic syndrome. The difference in gender persists. In females, the RRs of the middle and high score groups were 0.47 (RR = 0.47, 95% CI 0.23–0.96) and 0.21 (RR = 0.21, 95% CI 0.09–0.46), respectively. In males, by contrast, the protective effect of a healthy lifestyle was more pronounced in the high score group (RR = 0.33, 95% CI 0.13–0.83) and in females, the protective effects were found to be more likely. The protective effect of a healthy lifestyle on mortality was more pronounced in those aged < 65 years. Higher lifestyle scores were associated with more prominent protective effects, regardless of the presence of one metabolic syndrome factor or a combination of several factors in 15 groups. What's more, the protective effect of an emerging healthy lifestyle was more pronounced than that of a conventional lifestyle.

**Conclusions:**

Adherence to an emerging healthy lifestyle can reduce the risk of all-cause mortality in people with metabolic syndrome and metabolic syndrome-like characteristics; the higher the score, the more obvious the protective effect. Our study highlights lifestyle modification as a highly effective nonpharmacological approach that deserves further generalization.

**Supplementary Information:**

The online version contains supplementary material available at 10.1186/s12967-023-04062-1.

## Introduction

Chronic diseases are contributing to an increasing global disease burden. In recent years, metabolic syndrome (MetS) has become a major global health problem and has received much attention, as it is closely related to various mortality outcomes [1, 2]. The first formal definition of metabolic syndrome was developed by the World Health Organization (WHO) in 1998 [3]. The International Diabetes Federation (IDF) and American Heart Association/National Heart, Lung, and Blood Institute (AHA/NHLBI) reached a consensus on the definition of metabolic syndrome in recent years [4]. Metabolic syndrome is a group of disorders that include central obesity, elevated triglycerides, reduced high-density lipoprotein cholesterol (HDL-C), high blood pressure, and elevated fasting glucose; the presence of three or more of these five characteristics is called metabolic syndrome [4–6]. Each disease itself is a risk factor for other conditions. However, the combination of these diseases greatly increases the chance of developing potentially life-threatening conditions such as type 2 diabetes mellitus, cardiovascular disease (CVD), or stroke [7–9]. The health risks also increase when the number of components increases [7]. Metabolic syndrome typically begins with insulin resistance, and its pathogenesis involves oxidative stress and activation of the renin-angiotensin system [10]. High glucose can increase cardiotoxicity and reduce the effectiveness of anticancer drugs by activating the NLRP3 inflammasome and a large number of cytokines [11]. Metabolic syndrome is affected by a mixture of genetic and behavioral factors, and lifestyle interventions are often ignored in favor of medication [5, 12]. Nevertheless, lifestyle changes can be a simple and achievable way to delay the development of a disease.

Lifestyle factors can cause several chronic diseases. Accumulating evidence suggests that the management of a healthy lifestyle is becoming increasingly important [13, 14]. Conventional modifiable lifestyle factors, namely no current smoking, moderate alcohol consumption, regular physical activity, and a healthy diet, have received sufficient attention in previous studies [15–17]. However, new lifestyle factors have also been discussed, such as less sedentary behavior and adequate sleep duration [18, 19]. There have been many studies on the relationship between conventional lifestyles and mortality. However, evidence of the combination of conventional and emerging lifestyles is lacking, especially in people with metabolic syndrome and metabolic syndrome-like characteristics. Multiple studies have focused on the lifestyle factors of the general population or even specific populations, such as people with type 2 diabetes or neurodegenerative diseases [18, 20]. With regard to people with multiple comorbidities of metabolic syndrome, this area of research remains largely unexplored.

To fill this knowledge gap, we included participants from the National Health and Nutrition Examination Survey (NHANES) to examine whether six newly emerging healthy lifestyles could reduce the risk of all-cause mortality in people with metabolic syndrome and those with metabolic syndrome-like characteristics. We also wanted to determine how much a healthy lifestyle might affect outcomes. Furthermore, we wondered whether the effect of adherence to a relatively low-risk lifestyle on mortality varied depending on different combinations of metabolic syndrome characteristics.

## Methods

### Study population

The NHANES is a major program of the National Center for Health Statistics (NCHS). It was designed to assess the health and nutritional status of adults and children in the United States. Written informed consent was obtained from all the participants. It began in the early 1960s and became a continuous program in 1999. It combines interviews and physical examinations and is based on a two-year survey cycle. The survey is conducted annually on a nationally representative sample of approximately 5000 people and oversampled for specific age and ethnic groups. Patient identifiers are not available in the publicly available NHANES database.

**Fig. 1:**
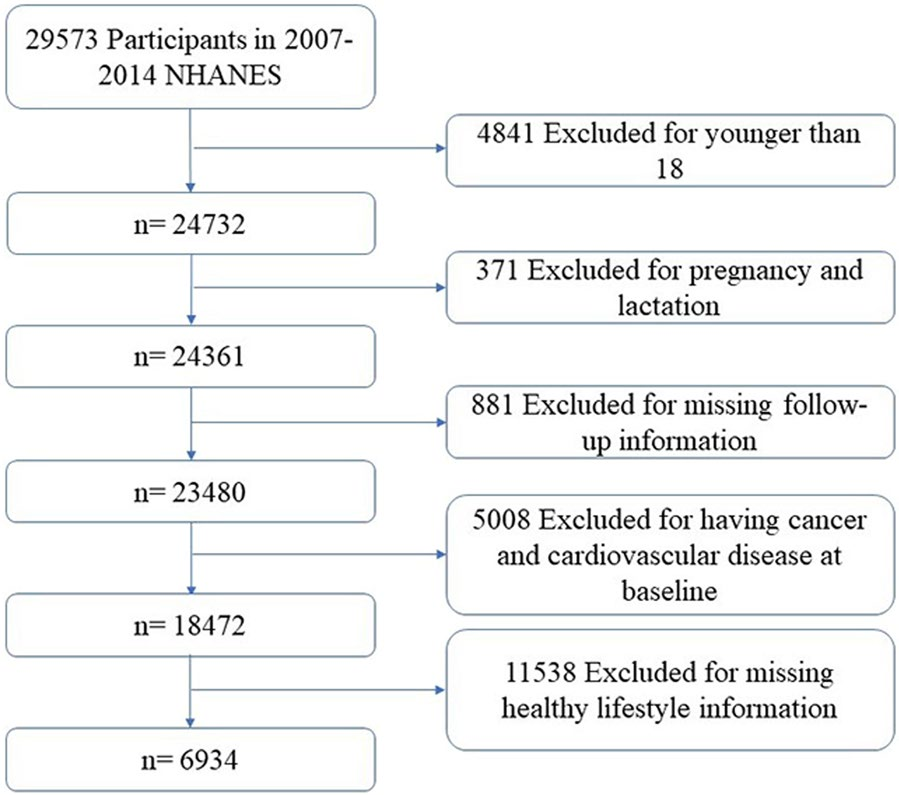
Flow chart of the screening of eligible participants

A total of 29,573 participants from the NHANES 2007–2014 were included in the study. Of these 29,573 participants, we excluded those who were younger than 18 years of age (n = 4841), pregnant or lactating (n = 371), had unreliable or missing follow-up information (n = 881), had cancer or cardiovascular disease at baseline (n = 5008), and had missing values on lifestyle scores (n = 11,538), leaving 6934 participants in the final analysis (Fig. 1).

Metabolic syndrome refers to a group of diseases with three or more of the following features, including central obesity, elevated triglycerides, reduced high-density lipoprotein cholesterol, high blood pressure, and elevated fasting glucose. High triglycerides and reduced HDL levels are collectively referred to as abnormal lipid metabolism. In addition, we grouped metabolic syndrome into four essential features: central obesity, dyslipidemia, hypertension, and hyperglycemia. We further divided the participants into those with metabolic syndrome and 15 groups with metabolic syndrome characteristics. We performed possible combinations based on the four main features of the metabolic syndrome, including those of any one feature (central obesity group, dyslipidemia group, hypertension group, and hyperglycemia group), combinations of two features (central obesity and dyslipidemia group, central obesity and hypertension group, central obesity and hyperglycemia group, dyslipidemia and hypertension group, dyslipidemia and hyperglycemia group, hypertension and hyperglycemia group), combinations of three features (central obesity, dyslipidemia and hypertension group, central obesity, dyslipidemia, and hyperglycemia group, central obesity, hypertension, and hyperglycemia group, dyslipidemia, hypertension, and hyperglycemia group), and all four features (central obesity, dyslipidemia, hypertension, and hyperglycemia group), for a total of 15 groups.

### Healthy lifestyle score

Information on the lifestyles of participants was obtained using self-reported questionnaires. In this study, in addition to conventional healthy lifestyle factors (no smoking, moderate alcohol consumption, moderate to vigorous physical activity, and high-quality diet), we incorporated emerging behavioral factors (sleep duration and sedentary behavior) [21, 22]. A detailed definition of a healthy lifestyle is provided in the Additional file 1 [16–18, 23–29].

We assigned a score of one to a healthy lifestyle and zero to an unhealthy lifestyle. To better reflect the effect of each healthy lifestyle factor on the outcome, we constructed a healthy lifestyle score by calculating a weighted healthy lifestyle score [30]. To avoid extreme groups, we divided the lifestyle scores into three groups, which were divided into thirds.

### Covariates

Socioeconomic factor-related covariates were mainly obtained from home interviews in the form of questionnaires. They included age, sex, ethnicity, education, and the income-to-poverty ratio (PIR). Ethnic groups were mainly divided into five groups: Mexican Americans, other Hispanics, non-Hispanic white, non-Hispanic black, and other races. Education was categorized into less than high school diploma, high school graduate or equivalent, some college or associate degree, and college or above. The PIR was divided into three groups based on the following criteria: ≤ 1.0, PIR > 1.0, PIR ≤ 3.0, and PIR > 3.0.

Other covariates included the laboratory data. Blood specimens were processed, stored, and transported to the Fairview Medical Center Laboratory at the University of Minnesota, Minneapolis, MN, USA, for analysis. The staff were well trained, and the NCHS developed and distributed a quality control protocol to each NHANES contract laboratory. Because the criteria for characterizing metabolic syndrome included values for triglycerides and HDL, we did not include either indicator in our model.

### Assessment of the outcomes

The NCHS links data collected by population surveys to death certificate records in the National Death Index (NDI). Public use linked mortality files (LMF) includes a limited set of variables, applies only to adult participants, and is subject to data perturbation techniques to reduce the risk of participant disclosure. The publicly available LMF includes follow-up data on mortality from the date the participants entered the survey until December 31, 2019, which is the most recent data available. The data released for public use include the addition of perturbative data for two elements: the date of death and the cause of death. For more information on accessing the restricted use linked mortality files, please refer to the official website (https://www.cdc.gov/nchs/data-linkage). The National Database of Death Statistics is a central database of death record information archived in judicial vital records or statistical offices and maintained by the National Center for Vital Statistics. Participants were deemed eligible for mortality follow-up if sufficient information was provided during the interview or at the mobile examination center (MEC) follow-up. These data can be used to determine the cause and manner of death for each person who died. Basic and multiple causes of death were classified according to the International Classification of Diseases, Ninth Revision (ICD-9), and Tenth Revision (ICD-10) since 1999. This study defined the outcome as all-cause mortality (heart disease, cancer, chronic lower respiratory disease, unintentional injuries, and cerebrovascular diseases).

### Statistical analysis

Data were analyzed according to the guidelines provided by the NHANES. Baseline characteristic variables were expressed as n (%) if they were categorical, as the mean (SD) for normally distributed variables, or as the median (interquartile range) for non-normally distributed variables. The study population was divided into three groups according to lifestyle scores for between-group analysis. The Chi-square test, ANOVA, and Kruskal–Wallis test were used to compare the distribution of baseline characteristics among the different lifestyle score groups [18].

Generalized linear regression models and restricted cubic splines were used to analyze the association between healthy lifestyle scores and all-cause mortality risk [31]. The results were presented as risk ratios (RRs) with 95% confidence intervals (CI). Three regression models were constructed to account for the influence of confounding factors: model 1 included age and sex; model 2 additionally included ethnicity, education, and PIR; and model 3 additionally included HbA1c% levels. According to the weighted healthy lifestyle score, patients were divided into three levels: low, middle, and high. Age (< 65 years and ≥ 65 years) and sex stratification analyses were performed. We also examined the association between healthy lifestyle scores and all-cause mortality among participants with metabolic syndrome. Based on the aforementioned grouping, we examined different combinations of the basic characteristics of total metabolic function and a population with all the basic characteristics so that 15 groups were included in the analysis.

Sensitivity analyses were also conducted. We calculated the conventional healthy lifestyle score and compared it with the emerging healthy lifestyle score in the overall population, males, and females. Data analysis was performed using STATA 16, and *p* < 0.05 was considered statistically significant [32].

## Results

### Characteristics of the participants

A total of 6934 participants were enrolled in this study. In total, 295 deaths were recorded. Based on their weighted healthy lifestyle scores, they were classified into three groups: low (n = 2561, 36.93%), middle (n = 2076, 29.94%), and high (n = 2297, 33.13%). The baseline characteristics of the study participants are shown in Table 1. In contrast to the low lifestyle score group, the high lifestyle score group had a greater proportion of older people. In terms of population distribution, non-Hispanic whites accounted for the largest proportion. The lowest PIR was found in the group with the lowest healthy lifestyle score, indicating the highest level of poverty. The proportion of people with a college degree or above was the lowest in the low lifestyle score group. With an increase in the score, the proportion of smokers decreased. There were no current smokers in the high score group. Changes in diet and sedentary behavior among the three groups were most pronounced as the scores increased.

**Table 1:** Baseline characteristics of the participants

	Lifestyle score			
	Low (n = 2561)	Middle (n = 2076)	High (n = 2297)	*p*
Age, years	36.0 [27.0, 49.0]	44.0 [31.0, 58.0]	45.0 [32.0, 60.0]	**< 0.001**^**b**^
< 65y	2396 (93.6%)	1772 (85.4%)	1908 (83.1%)	
≥ 65y	165 (6.4%)	304 (14.6%)	389 (16.9%)	
Gender				**< 0.001**^**a**^
Men	1450 (56.6%)	987 (47.5%)	1131 (49.2%)	
Women	1111 (43.4%)	1089 (52.5%)	1166 (50.8%)	
Ethnicity				**< 0.001**^**a**^
Mexican American	261 (10.2%)	210 (10.1%)	390 (17.0%)	
Other Hispanic	211 (8.2%)	164 (7.9%)	265 (11.5%)	
Non-Hispanic White	1236 (48.3%)	966 (46.5%)	993 (43.2%)	
Non-Hispanic Black	604 (23.6%)	414 (20.0%)	366 (15.9%)	
Other race	249 (9.7%)	322 (15.5%)	283 (12.3%)	
PIR	2.4 [1.2, 4.5]	3.5 [1.7, 5.0]	2.7 [1.3, 5.0]	**< 0.001**^**b**^
Education				**< 0.001**^**a**^
Less than high school diploma	372 (14.5%)	188 (9.1%)	393 (17.1%)	
High school graduate or equivalent	613 (23.9%)	319 (15.4%)	434 (18.9%)	
Some college or associates degree	927 (36.2%)	637 (30.7%)	686 (29.9%)	
College or above	646 (25.2%)	931 (44.8%)	783 (34.1%)	
Smoking				**< 0.001**^**a**^
Never	1281 (50.0%)	1489 (71.7%)	1755 (76.4%)	
Former	407 (15.9%)	519 (25.0%)	542 (23.6%)	
Current	873 (34.1%)	68 (3.3%)	0 (0%)	
Alcohol consumption				**< 0.001**^**a**^
Binge drinking	1485 (58.0%)	791 (38.1%)	549 (23.9%)	
Non-binge drinking	1076 (42.0%)	1285 (61.9%)	1748 (76.1%)	
Healthy diet	172 (6.7%)	1597 (76.9%)	1540 (67.0%)	**< 0.001**^**a**^
Regular physical activity	1589 (62.0%)	1364 (65.7%)	1864 (81.1%)	**< 0.001**^**a**^
Adequate sleep duration	1159 (42.3%)	1185 (57.1%)	1656 (72.1%)	**< 0.001**^**a**^
Less sedentary behaviors	225 (8.8%)	115 (5.5%)	1581 (68.8%)	**< 0.001**^**a**^
LDL-cholesterol (mg/dL)	112.0 [90.0, 138.0]	111.0 [91.0, 132.0]	112.0 [90.0, 137.0]	0.584^b^
HDL-cholesterol (mg/dL)	49.0 [40.0, 60.0]	54.0 [44.0, 65.0]	53.0 [44.0, 65.0]	**< 0.001**^**b**^
Triglycerides (mg/dL)	119.0[76.5, 186.0]	110.0 [75.0, 171.0]	108.0 [72.0, 168.0]	**< 0.001**^**b**^
HbA1c%	5.4 [5.1, 5.7]	5.4 [5.1, 5.7]	5.4 [5.2, 5.7]	**< 0.001**^**b**^

PIR: family income to poverty ratio; LDL: low-density lipoprotein; HDL: high-density lipoprotein; HbA1c: glycated hemoglobin. *p*-values in bold indicate statistical significance. ^a^Represents the use of the Chi-square test. ^b^Represents the use of the Kruskal–Wallis test

### Association of emerging healthy lifestyle factors and all-cause mortality

During the follow-up, 129 deaths were recorded. Based on the definition of metabolic syndrome, we finally included 1697 individuals for analysis to explore the association of the weighted healthy lifestyle score with all-cause mortality by performing generalized linear regression analyses. As shown in Table 2, the relative risk of all-cause mortality decreased as the lifestyle score increased. In model 1, after adjusting for age and sex, compared with participants with a relatively low healthy lifestyle, the RR of the middle group was 0.51 (RR = 0.51, 95% CI 0.30–0.88), and the high group was 0.26 (RR = 0.26, 95% CI 0.15–0.48). In model 2, adjusted for age, sex, ethnicity, education, and PIR, the RR was 0.55 (RR = 0.55, 95% CI 0.32–0.94) for the middle group and 0.27 (RR = 0.27, 95% CI 0.15–0.48) for the high score group compared with the low score group. And in model 3, after adjusting additionally included HbA1c% levels, the multivariate-adjusted RR of the middle group was 0.56 (RR = 0.56, 95% CI 0.33–0.97), and the high group was 0.26 (RR = 0.26, 95% CI 0.14–0.47).

**Table 2:** RRs (95% CI) of all-cause mortality according to lifestyle category, age, and sex

	Risk ratio (95% CI)		
	Model 1	Model 2	Model 3
The overall population			
Low lifestyle score	1 (Reference)	1 (Reference)	1 (Reference)
Middle lifestyle score	0.51 (0.30, 0.88)*	0.55 (0.32, 0.94)*	0.56 (0.33, 0.97)*
High lifestyle score	0.26 (0.15, 0.48)*	0.27 (0.15, 0.48)*	0.26 (0.14, 0.47)*
Male			
Low lifestyle score	1 (Reference)	1 (Reference)	1 (Reference)
Middle lifestyle score	0.59 (0.24, 1.45)	0.61 (0.26, 1.41)	0.61 (0.26, 1.40)
High lifestyle score	0.33 (0.13, 0.83)*	0.35 (0.14, 0.88)*	0.33 (0.13, 0.85)*
Female			
Low lifestyle score	1 (Reference)	1 (Reference)	1 (Reference)
Middle lifestyle score	0.47 (0.23, 0.96)*	0.50 (0.24, 1.03)	0.52 (0.25, 1.08)
High lifestyle score	0.21 (0.09, 0.46)*	0.21 (0.09, 0.47)*	0.19 (0.08, 0.44)*
By age group			
< 65 years old			
Low lifestyle score	1 (Reference)	1 (Reference)	1 (Reference)
Middle lifestyle score	0.34 (0.12, 0.98)*	0.34 (0.12, 1.00)*	0.35 (0.12, 1.03)
High lifestyle score	0.06 (0.01, 0.26)*	0.06 (0.01, 0.27)*	0.06 (0.01, 0.29)*
≥ 65 years old			
Low lifestyle score	1 (Reference)	1 (Reference)	1 (Reference)
Middle lifestyle score	0.70 (0.37, 1.34)	0.71 (0.38, 1.33)	0.73 (0.39, 1.36)
High lifestyle score	0.45 (0.22, 0.91)*	0.43 (0.21, 0.89)*	0.41 (0.20, 0.87)*

CI: confidence interval. The asterisk indicates statistical significance

### Subgroup analyses

Similar results were obtained in stratified analyses by sex and age. The impact of a healthy lifestyle on the relative risk of mortality from any cause is more pronounced among females. As shown in Table 2, in females, the RRs of the middle score group and the high score group were 0.47 (RR = 0.47, 95% CI 0.23–0.96) and 0.21 (RR = 0.21, 95% CI 0.09–0.46), respectively, when compared with the low score group in model 1. When additional covariates were adjusted, only the RRs of the high group were statistically significant, with 0.21 (RR = 0.21, 95% CI 0.09–0.47) in model 2 and 0.19 (RR = 0.19, 95% CI 0.08–0.44) in model 3. In the analysis performed with males, the protective effect of a healthy lifestyle was more pronounced only in the high score group (model 1: RR = 0.33, 95% CI 0.13–0.83, model 2: RR = 0.35, 95% CI 0.14–0.88, model 3: RR = 0.33, 95% CI 0.13–0.85). We divided the participants into two groups according to whether they were under or over 65 years old and performed stratified analyses. The protective effect of a healthy lifestyle on mortality was more evident in people younger than 65 years than in those older than 65 years. Moreover, the older the age, the higher the lifestyle score required to play a significant protective role.

### Association of emerging healthy lifestyle factors and all-cause mortality in people with metabolic syndrome-like characteristics

**Fig. 2:**
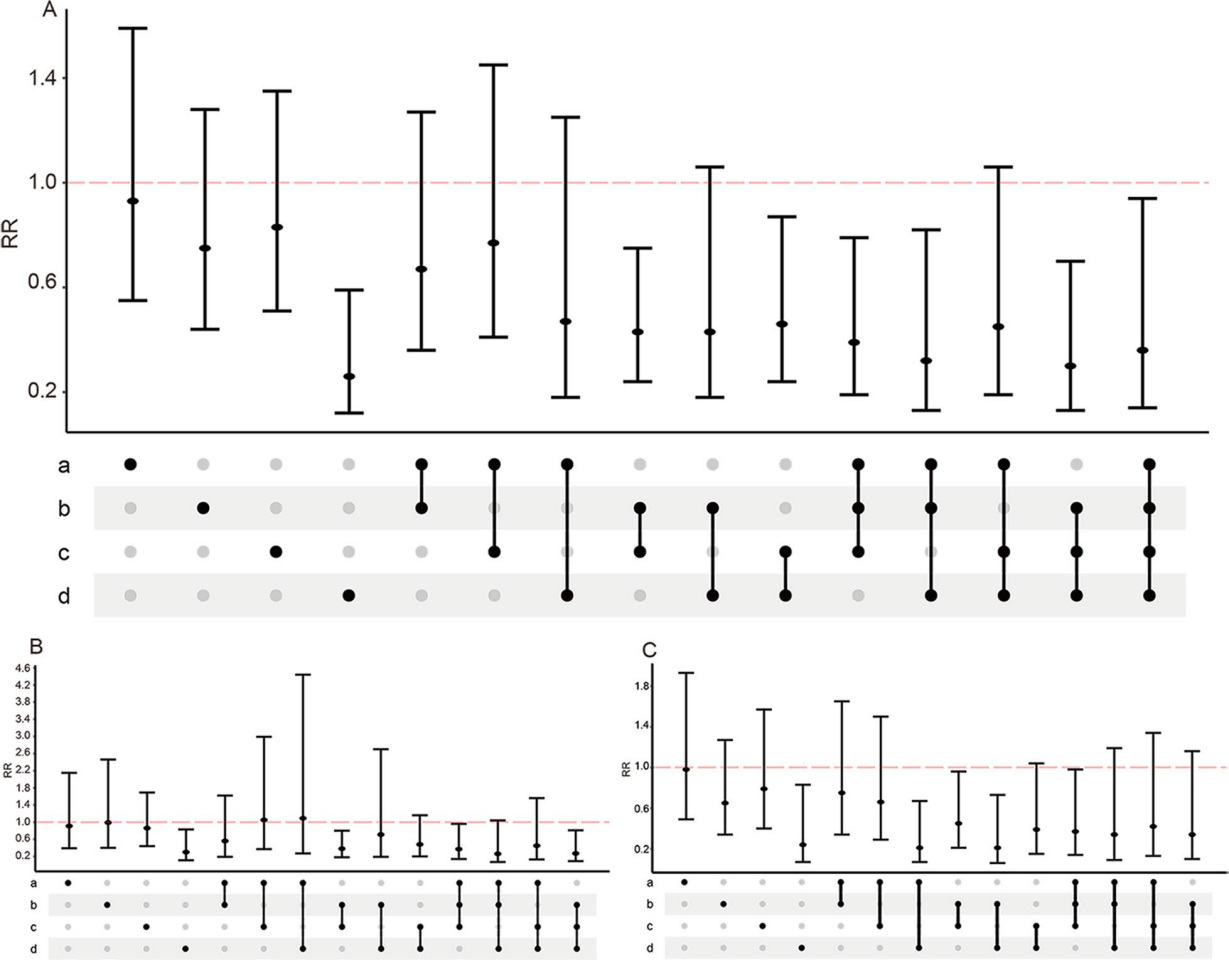
Relationship between participants with metabolic syndrome-like characteristics and all-cause mortality in the middle score group. **A** RRs in all population; **B** RRs in males; **C** RRs in females; a represents the characteristics of central obesity, b represents the characteristics of dyslipidemia, c represents the characteristics of hypertension, and d represents the characteristics of hyperglycemia

**Fig. 3:**
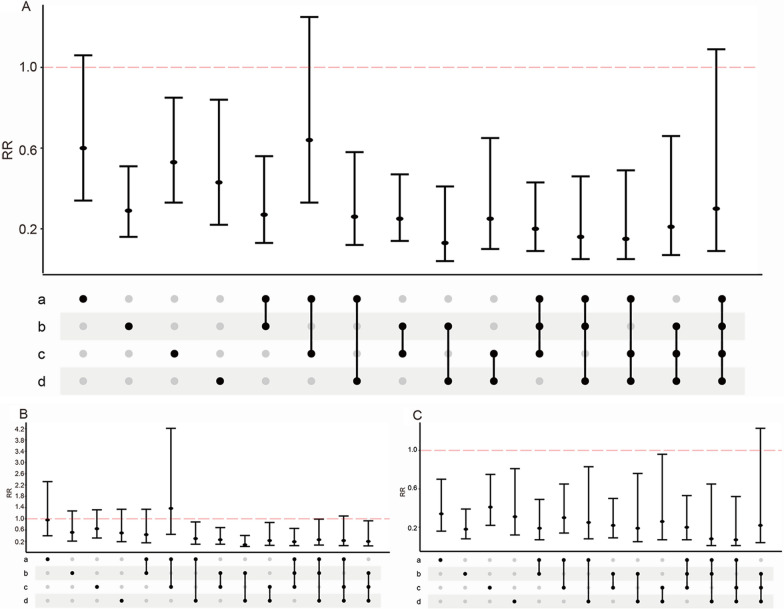
Relationship between participants with metabolic syndrome-like characteristics and all-cause mortality in the high score group. **A** RRs in all population; **B** RRs in males; **C** RRs in females; a represents the characteristics of central obesity, b represents the characteristics of dyslipidemia, c represents the characteristics of hypertension, and d represents the characteristics of hyperglycemia

Participants with features of metabolic syndrome, that is, one of the following four features or a combination of two, three, or four of them, were included in the analysis. We performed population-wide and sex-stratified analyses within these 15 groups. As shown in Figs. 2 and 3, in general, a higher lifestyle score was associated with a more obvious protective effect, regardless of the presence of one of the metabolic syndrome factors or a combination of several factors, and this was true for both males and females. In the dyslipidemia and hypertension groups, only the high score group had a reduced risk of all-cause mortality, with an RR of 0.29 (RR = 0.29, 95% CI 0.16–0.51) and 0.53 (RR = 0.53, 95% CI 0.33–0.85), respectively. The middle and high lifestyle scores were not statistically significant in the central obesity group. Interestingly, in the hyperglycemia group, the RR was higher in the high score group (RR = 0.43, 95% CI 0.22–0.84) than in the middle score group (RR = 0.26, 95% CI 0.12–0.59). In most cases, it was found to be easier for female patients to achieve meaningful results. There was no statistically significant difference in the group of patients with central obesity and hypertension when the patients had two features of metabolic syndrome. When the patients had three characteristics of metabolic syndrome, the high score group had a reduced RR compared with the middle and low score groups.

**Fig. 4:**
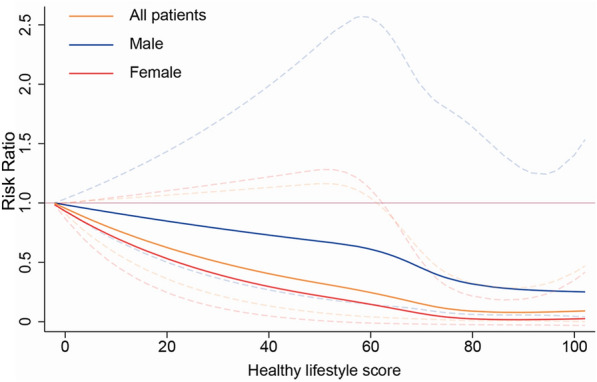
Dose–response relationship between weighted healthy lifestyle scores and all-cause mortality. The solid and dashed lines indicate the estimated RRs and their 95% confidence intervals

The dose–response relationship between a healthy lifestyle and all-cause mortality is shown in Fig. 4. In general, the healthy lifestyle score showed a nonlinear negative correlation with all-cause mortality with a relatively flat curve. As the score increased, the risk of all-cause mortality decreased. This negative nonlinear relationship became significant in all populations when the score exceeded 61.6. We further performed dose–response analyses for both males and females. Among females, a score above 63.7 was needed for a negative relationship to become statistically significant, whereas the relationship was not significant among males.

### Sensitivity analyses

**Fig. 5:**
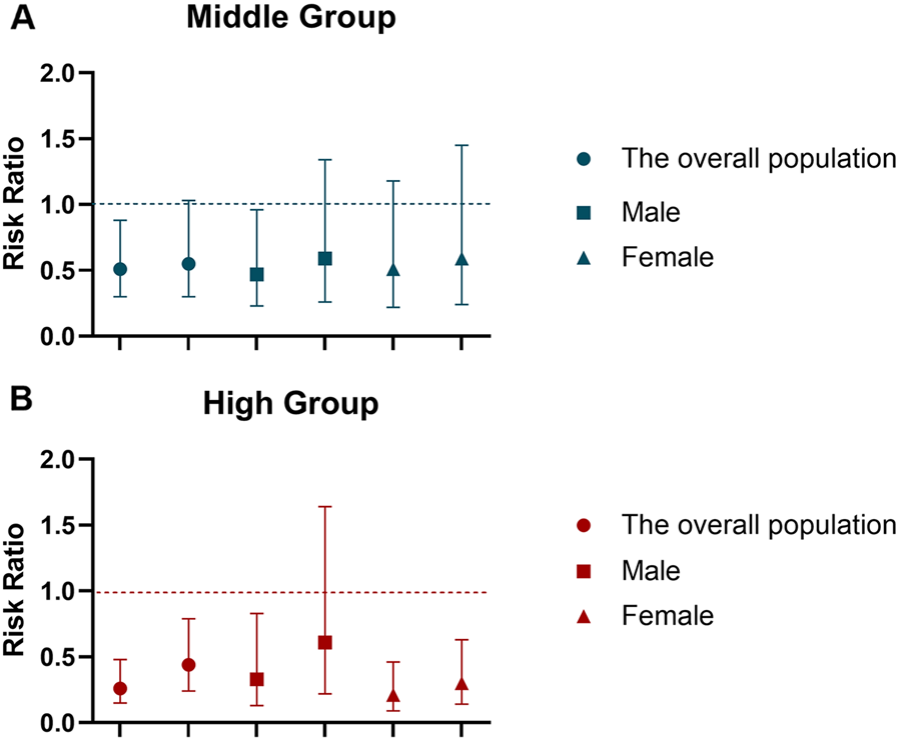
Comparison of the conventional and emerging healthy lifestyle scores. **A** Comparison in the middle score group; **B** comparison in the high score group. Of the two groups represented by the same legend, the one on the left represents the emerging healthy lifestyle group, and the one on the right represents the conventional healthy lifestyle group

The results did not change in the sensitivity analyses. The conventional lifestyle score reduced the risk of all-cause mortality among all the population with metabolic syndrome, particularly with a significant effect in the group with a high score (RR = 0.44, 95% CI 0.24–0.79), and again, it was meaningful among females. In addition, the protective effect of the newly emerging healthy lifestyle factors was stronger, and the RR was lower than that of the conventional lifestyle factors, both in the whole population and in females (Fig. 5).

## Discussion

In this prospective cohort study, we investigated the association between lifestyle scores based on six low-risk lifestyle factors and all-cause mortality in patients with metabolic syndrome and metabolic syndrome-like characteristics. A weighted score was constructed to reflect the effect of each lifestyle factor on the outcome. As a result, compared with the low-weighted score group, the middle group had a reduced risk of all-cause mortality, and the high group had a further reduced risk regardless of socioeconomic factors and biochemical indicators. We found consistent results when sex- and age-stratified analyses were performed. To the best of our knowledge, this is the first prospective study to explore the relationship between emerging healthy lifestyles and all-cause mortality in patients with metabolic syndrome. Our study adds to this field by examining the impact of multiple behavior-related risk factors on health and simultaneously shows that the findings are relatively consistent with those of previous studies. This study sheds new light on health management and primary prevention in populations with metabolic syndrome and metabolic syndrome-like characteristics.

A healthy lifestyle reduces the burden of disease and prolongs the life span of the population [33, 34]. People with higher healthy lifestyle scores were more likely to have higher levels of education and lower poverty, indicating that a healthy lifestyle is influenced by many economic and social factors. People with high scores tend to be older, possibly because they have more time to pay attention and improve their lifestyles. The lifestyle behaviors that differed most across groups were diet, sedentary behavior, and sleep duration, which may require longer adherence than other lifestyle behaviors. In the three models that we constructed, the protective effect of a healthy lifestyle persisted as the number of covariates increased. Studies on conventional healthy lifestyles have been previously conducted. Our study is consistent with the results of previous cohort studies conducted in the United States and the United Kingdom [16–18, 30, 35]. For example, Li et al. showed that adherence to a healthy lifestyle was associated with a longer life expectancy free of major chronic diseases in two cohorts from the United States [16]. In a study by Zhang et al., a healthy lifestyle was shown to be associated with a lower risk of mortality and cardiovascular disease burden in two large cohorts, one from the United States and one from the United Kingdom [17]. Consistent with previous studies, a low-risk lifestyle was associated with a protective effect against mortality, with RRs further decreasing as the weighted healthy lifestyle score increased.

Additionally, the association between a healthy lifestyle and mortality has been studied in different populations. Chudasama et al. showed that adherence to a healthy lifestyle could improve life expectancy in people with multimorbidity [30]. In individuals with type 2 diabetes, a low-risk lifestyle was also found to be protective [18]. In some central nervous system diseases such as stroke, previous studies have shown similar conclusions [20]. Dhana et al. reported that a comprehensive lifestyle is associated with a lower risk of developing Alzheimer’s disease [36]. A favorable lifestyle is associated with a lower risk of developing dementia even in people with a high genetic risk [37]. Recent epidemiological studies have highlighted the significance of comprehensive dietary patterns rather than single nutritional elements [38]. Study has shown that a proper diet can regulate metabolism through a variety of ways and has anti-inflammatory effects, and can significantly reduce the incidence of metabolic syndrome [39]. In this study, we adopted the widely used HEI-2015 to integrally reflect the intake of various nutritional elements. Recent studies have shown that sleep patterns can reduce the risk of all-cause and cause-specific mortality in several large cohort studies [28, 40, 41]. In addition to sleep, increasing attention has been paid to the evaluation of sedentary behavior in a composite healthy lifestyle. Less sedentary behavior is related to a reduced risk of premature mortality and cardiovascular disease mortality [42, 43]. On this basis, studies that include conventional healthy lifestyle factors in addition to emerging healthy lifestyle factors are rare [18]. The sensitivity analysis showed that the six new healthy lifestyle factors had a more significant protective effect on mortality outcomes than the four conventional healthy lifestyle factors.

Dietary intake information on individual food intake is available in NHANES. However, it is usually the 24-h dietary recall of total nutrient intake that is widely used in research because it is convenient and easy to calculate [27]. Some studies calculated participants' exercise levels by wearing an accelerometer [44]. But relatively few people obtained the amount of exercise by this method, so we chose the variable with the largest number of participants, the metabolic equivalent of the task. For example, only participants from 2011 to 2014 were tested for body movement by wearing accelerators. However, we had access to questionnaire information for participants from 2007 to 2014. Previous studies have defined “never smoking” as a healthy level [16]. However, we believe that this criterion is somewhat harsh. Therefore, in this study, we define “no current smoking” as a healthy level. There are also studies that use “never drinking” as a healthy standard [45]. We believe that people will inevitably drink alcohol throughout their lives, so we choose “non-heavy drinking” as a standard of health.

With the introduction of the concept of metabolic syndrome, it has attracted increasing attention. There are a considerable number of patients with metabolic syndrome. Patients with chronic diseases are more likely to have metabolic syndrome [46]. Moreover, when patients have metabolic syndrome or metabolic syndrome-like characteristics in combination with other underlying conditions, such as heart disease and cancer, the risk of death is increased [11]. In vitro studies have confirmed that high glucose, one of the characteristics of metabolic syndrome, increases cardiotoxicity and reduces the effectiveness of anticancer drugs through inflammatory pathways, and that inflammatory factors play an important role in heart failure in cancer patients [11]. However, few relevant studies on the primary prevention of metabolic syndrome have been conducted. The treatment of metabolic syndrome needs to be individualized, especially pharmacotherapy. Lifestyle modification is a relatively simple treatment without drug side effects. In this study, we concluded that a high lifestyle score is associated with a reduced risk of all-cause mortality in people with metabolic syndrome and those with features of metabolic syndrome. Compared to people with central obesity, those with dyslipidemia and hypertension could significantly reduce their risk of all-cause mortality through a favorable lifestyle, especially those with dyslipidemia. Among those with more than two features of metabolic syndrome, those with central obesity required a higher lifestyle score to reduce the risk of all-cause mortality. In people with metabolic syndrome, the proportion of patients with central obesity has been reported to be > 80% [5, 47]. The relationships between central obesity and other metabolic risk factors are different and they interact with each other. Most researchers agree that obesity is influenced by epigenetic, genetic, and lifestyle factors [48, 49]. Therefore, we speculate that patients with central obesity should adopt a healthier lifestyle to reduce the interference of epigenetic and genetic factors. Curiously, we observed that in the hyperglycemic group, a high healthy lifestyle score was associated with a higher risk ratio. The management of diabetes relies on self-management as well as medication [50]. Additionally, the severity of diabetes and medication use can affect the impact of a healthy lifestyle. Because a healthy lifestyle requires long-term adherence, we reasoned that the effects of a healthy lifestyle may become apparent over time with a longer follow-up period. Although the protective effect of high lifestyle scores was observed in males and females, the differences between males and females persisted. Our results showed that females appeared to be more strongly associated with a lower risk of death associated with a healthy lifestyle, similar to previous studies [16, 51, 52]. However, the specific reason for this remains unclear.

The strengths of our study include rich resource information and a long and reliable follow-up, which made it possible to perform a full analysis of mortality. The NHANES provides detailed and repeated measures of relevant lifestyle factors, which makes our results sufficiently dependable. Our results were further improved and confirmed after adjusting for socioeconomic factors and biochemical indicators using a multivariate covariate adjusted generalized linear regression model. Additionally, a series of sensitivity analyses made our results more robust. This study, based on a large population and a long follow-up period, helps people with metabolic syndrome and metabolic syndrome-like characteristics make the right choices. Our study also has several limitations, such as the number of subjects included and limited follow-up time. In addition, the target population of the NHANES is the non-institutionalized civilian resident of the United States; therefore, our findings may not be generalizable to other populations, and further research is needed.

## Conclusion

In aggregate, this study confirmed that a middle weighted healthy lifestyle score was associated with a lower risk of all-cause mortality than a low score, and that a high score was associated with further reduced risk among people with metabolic syndrome and those with features of metabolic syndrome. People with central obesity and other features of metabolic syndrome often require greater effort to adopt healthier lifestyles to achieve favorable outcomes. Moreover, the protective effect of the emerging healthy lifestyle factors was stronger than that of conventional healthy lifestyle factors, which is a notable finding. Our study provides a basis for further strengthening the management of patients with metabolic syndrome to reduce the global burden. A healthy lifestyle can be used as a simple nonpharmacological treatment without side effects and deserves to be promoted and popularized. In addition, our study suggests that future work should focus more on the management of people with central obesity. And efforts are needed to promote and disseminate adequate sleep duration and less sedentary behavior, two lifestyle factors that vary widely across populations.

## Supplementary Information

**Additional file 1: Table S1.** Detailed definition of a healthy lifestyle. **Table S2.** HEI–2015 Components and Scoring Standards.

## Data Availability

Data for this study are available at https://www.cdc.gov/nchs/nhanes/index.htm.

## Abbreviations

CI: Confidence intervals
CVD: Cardiovascular disease
HDL: High-density lipoprotein
HEI: Healthy eating index
LDL: Low-density lipoprotein
NCHS: National Center for Health Statistics
NHANES: National Health and Nutrition Examination Survey
PIR: Income-to-poverty ratio
RR: Risk ratio
