# Supplementary material for: Emerging healthy lifestyle factors and all-cause mortality among people with metabolic syndrome and metabolic syndrome-like characteristics in NHANES
Source: J Transl Med. 2023 Apr 1;21:239. doi: 10.1186/s12967-023-04062-1 (PMC10068159; doi:10.1186/s12967-023-04062-1)
Supplement: Supplementary file 1 — Additional file 1: Table S1. Detailed definition of a healthy lifestyle. Table S2. HEI–2015 Components and Scoring Standards. [file 12967_2023_4062_MOESM1_ESM.docx]

**Additional file 1**

**Emerging healthy lifestyle factors and all-cause mortality among people with metabolic syndrome and metabolic syndrome-like characteristics in NHANES**

**Short title: Emerging healthy lifestyle factors and all-cause mortality in people with MetS**

**Authors:** Mengying Niu, Jiahao Chen, Rongyao Hou, Yu Sun, Qi Xiao, Xudong Pan, Xiaoyan Zhu

**Experimental conditions：**

Participants completed alcohol questionnaires during a physical examination at the physical examination center using the Computer-Assisted Personal interview-CAPI system. Dietary intake data were used to estimate energy, nutrients, and other food components consumed in the 24 h preceding the interview. All NHANES participants underwent two 24-hour dietary recall interviews. The first dietary review interview was conducted in the mobile physical Examination Center (MEC), and the second interview was conducted by telephone 3 to 10 days later. As for smoking, physical activity, sleep duration, and sedentary activities, participants were asked these questions at home with the use of a Computer-Assisted Personal interview-CAPI system before a physical examination. ( https://www.cdc.gov/nchs/nhanes/index.htm)

**Table S1. Detailed definition of a healthy lifestyle:**

| **Lifestyle factors** | **Standard for 1 score** | **References** |
| --- | --- | --- |
| Smoking | No current smoking. | Han et al. 2022[1]. and Khera et al. 2016[2]. |
| Alcohol consumption | Less than 14 g of alcohol per day for females and less than 28 g for males. | Zhang et al. 2021[3]. and Song et al. 2021[4]. |
| Physical activity | At least 150 min of moderate activity, at least 75 min of vigorous activity per week, or an equivalent combination. | Han et al. 2022[1]. and Arnett et al. 2019[5]. |
| Diet | Top two-fifths of diet quality scores（(HEI)-2015）. | Li et al. 2020[6], Zhang et al. 2021[3], Smith et al. 2018[7] and Chen et al. 2019[8]. |
| Sleep | 7 and 8 h of sleep per day. | Han et al. 2022[1]. and Zhou et al. 2022[9]. |
| Sedentary behavior | Less than 4 h per day. | Han et al. 2022[1]. and Kelly et al. 2022[10]. |

**Table S2. HEI–2015 Components and Scoring Standards ^a^**

| HEI-2015 Components | Range of Points | Minimum Scoring Standard | Maximum Scoring Standard |
| --- | --- | --- | --- |
| **Adequacy Components** (higher score indicates higher consumption) | | | |
| Total Fruits | 0-5 | 0 | 0.8 cup equiv. /1000 kcal |
| Whole Fruits | 0-5 | 0 | 0.4 cup equiv./1000 kcal |
| Total Vegetables | 0-5 | 0 | 1.1 cup equiv. /1000 kcal |
| Greens and Beans | 0-5 | 0 | 0.2 cup equiv. /1000 kcal |
| Total Protein Foods | 0-5 | 0 | 2.5 oz equiv./1000 kcal |
| Seafood and Plant Proteins | 0-5 | 0 | 0.8 oz equiv./1000 kcal |
| Dairy | 0-10 | 0 | 1.3 cup equiv./1000 kcal |
| Whole Grains | 0-10 | 0 | 1.5 oz equiv. /1000 kcal |
| Fatty Acids ^b^ | 0-10 | (PUFAs + MUFAs)/SFAs ≤1.2 | (PUFAs + MUFAs)/SFAs ≥2.5 |
| **Moderation Components** (higher score indicates lower consumption) | | | |
| Refined Grains | 0-10 | 4.3 oz equiv./1000 kcal | 1.8 oz equiv. /1000 kcal |
| Sodium | 0-10 | 2.0 grams /1000 kcal | 1.1 grams/1000 kcal |
| Added Sugars | 0-10 | 26% of energy | 6.5% of energy |
| Saturated Fats | 0-10 | 16% of energy | 8% of energy |

^a^ Intakes between the minimum and maximum standards are scored proportionately.

^b^ Ratios of polyunsaturated and monounsaturated fatty acids (PUFAs and MUFAs) to saturated fatty acids (SFAs).

1. Han H, Cao Y, Feng C, Zheng Y, Dhana K, Zhu S, Shang C, Yuan C, Zong G: **Association of a Healthy Lifestyle With All-Cause and Cause-Specific Mortality Among Individuals With Type 2 Diabetes: A Prospective Study in UK Biobank.** *Diabetes Care* 2022, **45:**319-329.

2. Khera AV, Emdin CA, Drake I, Natarajan P, Bick AG, Cook NR, Chasman DI, Baber U, Mehran R, Rader DJ, et al: **Genetic Risk, Adherence to a Healthy Lifestyle, and Coronary Disease.** *N Engl J Med* 2016, **375:**2349-2358.

3. Zhang YB, Chen C, Pan XF, Guo J, Li Y, Franco OH, Liu G, Pan A: **Associations of healthy lifestyle and socioeconomic status with mortality and incident cardiovascular disease: two prospective cohort studies.** *BMJ* 2021, **373:**n604.

4. Song Z, Yang R, Wang W, Huang N, Zhuang Z, Han Y, Qi L, Xu M, Tang YD, Huang T: **Association of healthy lifestyle including a healthy sleep pattern with incident type 2 diabetes mellitus among individuals with hypertension.** *Cardiovasc Diabetol* 2021, **20:**239.

5. Arnett DK, Blumenthal RS, Albert MA, Buroker AB, Goldberger ZD, Hahn EJ, Himmelfarb CD, Khera A, Lloyd-Jones D, McEvoy JW, et al: **2019 ACC/AHA Guideline on the Primary Prevention of Cardiovascular Disease: Executive Summary: A Report of the American College of Cardiology/American Heart Association Task Force on Clinical Practice Guidelines.** *Circulation* 2019, **140:**e563-e595.

6. Li Y, Schoufour J, Wang DD, Dhana K, Pan A, Liu X, Song M, Liu G, Shin HJ, Sun Q, et al: **Healthy lifestyle and life expectancy free of cancer, cardiovascular disease, and type 2 diabetes: prospective cohort study.** *BMJ* 2020, **368:**l6669.

7. Krebs-Smith SM, Pannucci TE, Subar AF, Kirkpatrick SI, Lerman JL, Tooze JA, Wilson MM, Reedy J: **Update of the Healthy Eating Index: HEI-2015.** *J Acad Nutr Diet* 2018, **118:**1591-1602.

8. Chen F, Du M, Blumberg JB, Ho Chui KK, Ruan M, Rogers G, Shan Z, Zeng L, Zhang FF: **Association Among Dietary Supplement Use, Nutrient Intake, and Mortality Among U.S. Adults: A Cohort Study.** *Ann Intern Med* 2019, **170:**604-613.

9. Zhou T, Yuan Y, Xue Q, Li X, Wang M, Ma H, Heianza Y, Qi L: **Adherence to a healthy sleep pattern is associated with lower risks of all-cause, cardiovascular and cancer-specific mortality.** *J Intern Med* 2022, **291:**64-71.

10. Kelly NA, Soroka O, Onyebeke C, Pinheiro LC, Banerjee S, Safford MM, Goyal P: **Association of healthy lifestyle and all-cause mortality according to medication burden.** *J Am Geriatr Soc* 2022, **70:**415-428.
